# Sea conch (*Rapana venosa*) peptide hydrolysate regulates NF‐κB pathway and restores intestinal immune homeostasis in DSS‐induced colitis mice

**DOI:** 10.1002/fsn3.4410

**Published:** 2024-09-25

**Authors:** Hidayat Ullah, Yamina Alioui, Muhsin Ali, Sharafat Ali, Nabeel Ahmed Farooqui, Nimra Z. Siddiqui, Duaa M. Alsholi, Muhammad Ilyas, Mujeeb U. Rahman, Yi Xin, Liang Wang

**Affiliations:** ^1^ Department of Biotechnology, College of Basic Medical Science Dalian Medical University Dalian China; ^2^ Department of Biochemistry and Molecular Biology, College of Basic Medical Science Dalian Medical University Dalian China; ^3^ Stem Cell Clinical Research Center, National Joint Engineering Laboratory, Regenerative Medicine Center The First Affiliated Hospital of Dalian Medical University Dalian China

**Keywords:** FTIR, inflammatory bowel disease, intestinal immune homeostasis, peptides hydrolysate, scanning electronic microscopy

## Abstract

Inflammatory bowel disease (IBD) is a chronic inflammatory condition of the gastrointestinal tract. Sea conch peptide hydrolysate (CPH) was produced by enzymatic digestion of fresh conch meat with trypsin enzyme. To analyze the molecular composition, functional groups, and structural morphology of the hydrolysate, we employed liquid chromatography–mass spectrometry (LC–MS), Fourier‐transform infrared spectroscopy (FTIR), and scanning electron microscopy (SEM). Results confirmed that crude protein could be effectively digested by enzymes to generate peptides. In this study, we evaluated the bioactivities of CPH on dextran sulfate solution (DSS)‐induced colitis in mice. The findings demonstrated that CPH supplementation improved body weight, food and water intake, and colon length. The therapeutic efficacy and immunoregulatory effect of CPH were further determined. Our results exhibited that CPH treatment significantly ameliorated pathological symptoms by enhancing intestinal integrity, mucin production, and goblet cell count. Moreover, the immunoregulatory effect of CPH on mRNA expression levels of different pro‐ and anti‐inflammatory cytokines was determined. Results exhibited a decrease in the expression of pro‐inflammatory cytokines and an increase in anti‐inflammatory cytokines in the colon. Additionally, the CPH administration modulates the nuclear factor kappa B (NF‐κB) pathway, preventing DNA damage and cell death. Assays for apoptosis and DNA damage revealed that CPH reduced oxidative DNA damage and apoptosis. These findings highlight the immunomodulatory and treatment amelioration effect of CPH in reducing the severity of colitis.

## Graphical abstract

Conch peptides hydrolysate (CPH), derived from trypsin‐digested conch meat, was evaluated for its therapeutic effects on DSS‐induced colitis in mice. The study demonstrated that CPH improved clinical symptoms, enhanced intestinal integrity, and modulated immune responses by regulating cytokine levels and NF‐κB/TLR4 pathways, thereby reducing oxidative DNA damage and Apoptosis.

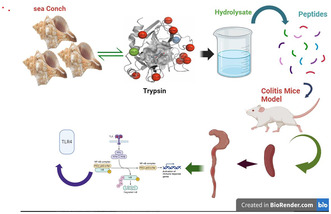

## 1 INTRODUCTION

Peptides are bioactive molecules consisting of 2–20 amino acids, linked through amide or peptide bonds (Sánchez & Vázquez, 2017). Ingested protein contains these peptides in an inactive form and is activated by metabolic processes, such as enzymatic hydrolysis, fermentation, and intestinal digestion inside the body (Chalamaiah et al., 2018). Peptides with lower molecular weight are easier to digest, exhibit higher activity, and have enhanced bioavailability compared to the original protein (Dziuba & Dziuba, 2014). Peptides have different biological activities, such as anti‐microbial properties, immunomodulatory, anti‐obesity, anti‐hypertensive, anti‐thrombotic, and anti‐carcinogenic activities, as well as antioxidant activity based on their amino acid composition and sequence (Chalamaiah et al., 2012; Ishak & Sarbon, 2018).

Fish‐derived bioactive peptides have demonstrated significant market value in the treatment of various diseases and health maintenance (Ishak & Sarbon, 2018). The molecular mass and amino acid sequence of the peptide play an important role in their bioactive properties (Halim et al., 2016). Research is focused on developing methods for the characterization and purification of these peptides, such as ultrafiltration, chromatography, gel filtration, and ion exchange chromatography (Vandanjon et al., 2007). Characterization of bioactive peptides is essential for the determination of amino acid sequence, its composition, and molecular weight. Qualitative methods like reversed‐phase high‐performance liquid chromatography (RP‐HPLC) and quantitative methods, such as automated amino acid analyzer, are used for amino acid composition analysis (Gu et al., 2011). Factors like enzyme source, fish raw material, and hydrolysis methods influence the composition. Additionally, mass spectroscopy, like MALDI (matrix‐assisted laser desorption) and FTIR (Fourier‐transform infrared spectroscopy), can be used to determine the amino acid sequence and molecular structure of peptides (Klompong et al., 2007).

The mammalian immune system consists of different types of immune cells and mediators that create a multifaceted network within the body, which helps protect the body from external pathogens while simultaneously maintaining immunity to self‐antigens (Belkaid & Hand, 2014; Zlott et al., 2017). Human health is reliant on the immune system, which directs a range of biological processes, monitors, and manages the defense system of the body to maintain homeostasis (Gao et al., 2019). Ulcerative colitis (UC) and Crohn's Disease (CD) are long‐term inflammatory diseases that primarily affect the gastrointestinal tract. UC and CD share common symptoms such as abdominal pain and diarrhea. UC is limited to only the colon while CD can involve both the colon and small bowel. In UC, the ulceration of the mucosal and submucosal layer causes the infiltration of immune cells. In CD, the inflammatory condition extends all layers of the bowel wall and may cause granulomas. Both conditions not only exhibit characteristics of chronic inflammation but also have an acute component with the influx of neutrophils, and both conditions have distinct features and patterns of inflammation (Macdermott & Stenson, 1988). The exact causes of UC and CD are still unknown. Different microbial and dietary factors have been proposed as potential etiologies, but none have been confirmed. The exact mechanism behind the unpredictable flare‐ups and remission characteristics of these diseases remains up for discussion. However, recent research highlights that the mechanism of the inflammatory response is intensified, resulting in the observed histology and clinical symptoms (Coskun, 2014; Mar et al., 2014).

Though, available medications for IBD include the use of anti‐inflammatory medicine, biological agents, anti‐diarrheal agents, aminosalicylates, antibiotics, and immune modulators (Baumgart & Sandborn, 2012; Engel & Neurath, 2010). However, these currently available treatments have drawbacks and can have detrimental effects on the immune system of patients. Long‐term use of these available medications can cause the patient to become more susceptible to infection and other illnesses (Marehbian et al., 2009; Van Assche et al., 2010).

Recently, the focus on studying functional foods has been gaining more attention including marine protein hydrolysates and bioactive peptides used for immunity improvement and disease treatment (Ambigaipalan & Shahidi, 2017). Research on natural active peptides has focused on a few marine sources. With the advancement in molecular modeling for drug discovery and peptide‐based cancer therapy research having become an exciting goal, marine sources offer substantial potential for drug development aimed at preventing and treating cancer. Since the 1980s, advancements in biotechnology have allowed researchers to recognize the marine environment as an important source for drug discovery and other applications.

*Rapana venosa*, generally known as sea conch a carnivorous sea snail, has become a popular seafood with dietary and economic benefits. *Rapana venosa* consumption positively affects lipid profile and antioxidant capacities (Merdzhanova et al., 2018). Hemocyanin from *R. venosa* shows an inhibitory effect on Epstein–Barr and herpes simplex virus (HSV) (Dolashka et al., 2014). *Rapana venosa* hemolymph yields proline‐rich peptides (Dolashka et al., 2011). Additionally, *R. venosa* is used in Oriental medicine for red eye ailments, liver heat, ophthalmalgia, chest pain, and abdominal pain (Benkendorff et al., 2015). These traditional uses highlight the significance and diverse health benefits of *R. venosa* for human health on human health.

The current study aims to characterize the sea conch peptide hydrolysate and its potential properties and to evaluate its immunomodulatory effects in a dextran sulfate sodium (DSS)‐induced colitis mouse model. We hypothesized that sea conch peptide hydrolysate possesses immunomodulatory properties that can alleviate colitis symptoms via regulating signaling pathways and restoring intestinal homeostasis.

## 2 MATERIALS AND METHODS

The sea snails were bought in Lüshunkou, Dalian, Liaoning, China, from a neighborhood seafood market. The supplier of trypsin, which was used in the digestion process, was RHAWN Chemicals, located in China. We acquired dextran sulfate sodium (DSS) from Yeasen Biotechnology, located in Shanghai China. Proteintech, a Chinese supplier with its headquarters in Wuhan, provided all of the antibodies used in this investigation. Shanghai Jianglai Industrial Share Ltd. provided the serum, interleukin 17 (IL‐17), tumor necrosis factor alpha (TNF‐α), interleukin 1 beta (IL‐1β), and interleukin 10 (IL‐10) enzyme‐linked immunosorbent assay (ELISA) kits. Thermo Fisher Scientific provided the Triazole reagents for this study, while typical commercial sources provided the remaining analytical‐grade reagents.

### 2.1 Sea conch peptides hydrolysate preparation

The enzymatic hydrolysis method for conch peptides hydrolysate preparation was utilized, as previously described by (Ullah et al., 2023). Briefly, the conch shell was removed, followed by mincing the meat using a grinder. The minced material underwent a washing process with double the volume of distilled water at 95°C for an hour. After soaking the meat residues for an hour, they underwent filtration through a 140‐μm sieve and were then combined with double the volume of distilled water. To aid in the enzymatic breakdown of conch meat, a 1% (w/w) concentration of trypsin enzyme was introduced into the mixture. This mixture was subsequently incubated at 50°C for 7 h, with continuous agitation maintained throughout. Following the 7‐h incubation period, enzyme activity was halted by subjecting the mixture to a temperature of 100°C for 20 min. The resultant solution underwent centrifugation at 14000 x *g* for 20 min at 4°C. The concentration of conch peptide hydrolysate (CPH) was determined using the Bradford method. Finally, the CPH supernatant was transformed into powder via a lyophilizer machine.

### 2.2 Molecular mass distribution and amino‐acid composition of CPH

To determine the molecular mass distribution and amino‐acid composition of different peptides in the hydrolysate of the conch, the hydrolysate underwent the technique of liquid chromatography–mass spectrometry (LC–MS). The mass range of m/z 200–2000 was used for the detection of precursor ions. The hydrolysate proteins and peptides were analyzed and identified from all collected data obtained from LC–MS.

### 2.3 Fourier‐transform infrared spectroscopy (FTIR) and scanning electron microscopy (SEM)

To analyze and characterize the peptide hydrolysate, the freeze‐dried sample was subjected to FTIR (Shimadzu FTIR‐4200 spectrometer) following (León‐López et al., 2019). The FTIR operated within the frequency range of 500–4000 cm^−1^. The analysis was performed at a scanning rate of 10 scans per second ensuring the resolution of 4 cm^−1^.

The morphological analysis was conducted using scanning electronic microscopy (SEM) (Model S‐2600N, Hitachi, Tokyo, Japan) to observe the shape and structural morphology of the peptide hydrolysate and crude sample (without enzyme digestion). Freeze‐dried powder of CPH and crude sample were affixed to strips of self‐adhesive carbon paper and coated with a thin layer of gold using a sputter coater. The samples were then examined under the scanning electronic microscope under an acceleration voltage of 15 kV.

### 2.4 Experimental design and animal accommodation

Fifty male BALB/c mice, aged 4–6 weeks, were utilized in this study, following approval from Dalian Medical University Ethical Committee under approval number 202310247. Prior to the experiment, the mice underwent a one‐week acclimatization period and were provided with unrestricted access to standard food and water. The experimental methodology was detailed in previous work (Ullah et al., 2023). Following the acclimatization phase, the mice were split into five groups: DSS group, Low dose CPH (LCP), Medium dose CPH (MCP), High dose CPH (HCP), and Normal Control (NC). For 7 days, 2.5% dextran sulfate sodium (DSS) was added to drinking water that had been autoclaved to cause colitis. On the 8th day, mice in the low, medium, and high dose groups were orally administered 100, 200, and 400 mg/kg of CPH, respectively, while mice in the NC and DSS groups received an equivalent volume of phosphate‐buffered saline (PBS) via oral gavage. After 22 days, stool samples were collected, and the mice were euthanized. The colon, small intestine, spleen, and other organs were harvested, fixed in 4% formalin, and stored at −80°C for subsequent analysis.

### 2.5 Body weight determination, Disease Activity Index measurement, food, and water consumption

Body weight was recorded daily throughout the experimental period following the incorporation of 2.5% DSS in water. Additionally, the food and water intake was determined by measuring it on every 3rd day. For the assessment of disease severity, the Disease Activity Index (DAI) is based on weight loss, diarrhea, and rectal bleeding as shown in (Table S1) following the protocol described by (Jeon et al., 2016). Weight loss was determined by the difference between initial and final weights. Diarrhea was defined as the absence of fecal pellets and the presence of continuous fluid stools. Rectal bleeding was assessed on visible blood in diarrhea and gross rectal bleeding. DAI was calculated as follows;

$$\mathrm{DAI}=\left(\text{weight loss score}+\text{diarrhea score}+\text{rectal bleeding score}\right)/3.$$

### 2.6 Histopathological examination of colon, small intestine, and spleen

Following the sacrificial protocol, the colon, spleen, and small intestine specimens were meticulously collected for subsequent analysis. To explore the histological modifications, microtome thin sections measuring 5 μm in thickness were meticulously prepared from the acquired tissues. These sections were then subjected to the hematoxylin–eosin (HE) staining technique following (Fischer et al., 2008) protocol. The tissue sections were first subjected to deparaffinization using fresh xylene for 10 min. Subsequently, the sections underwent rehydration through a series of ethanol gradients to restore their aqueous content. Thereafter, a hematoxylin–eosin staining protocol was executed on the tissue sections. A Lecia Microsystems microscope, manufactured in Wetzlar, Germany, was employed to observe and analyze the stained tissue section for pathological features in the colon, small intestine, and spleen.

### 2.7 Immunohistochemical and AB‐PAS staining for mucus in colon and small intestine

Immunohistochemistry was performed to determine the expression of Mucin‐2 (MUC2) in the ileum and colon. Five micrometer sections of paraffin‐embedded colon and small intestine tissue were prepared and placed on positively charged slides. The tissue sections were first subjected to deparaffinization using fresh xylene for 10 min and subsequent rehydration in decreasing ethanol gradient following the instruction given by the manufacturer. The data were analyzed using a semi‐quantitative approach. To determine whether immunolabeled cells were present, each slide was randomly examined under a microscope three times in various fields to assess the presence of immunolabeled cells. Alcian blue‐periodic acid–Schiff (AB‐PAS) staining was employed to assess neutral mucin, mucus epithelium thickness, and goblet cells in the colon. A 5‐μm‐thick section of colon tissue coated in paraffin was affixed onto slides. These slides underwent deparaffinization for 10 min in xylene. Subsequently, rehydration was carried out using a decreasing gradient of ethanol (e.g., 100%, 95%, 80%, 70%, and 50%) to remove any residual paraffin. Following the kit instructions, the slides were subjected to AB reagents and followed by three times washing with ultrapure water. PAS reagents were applied, and slides were incubated for 10 min. The slides were then washed with running tap water for 10 min and then rehydrated in ethanol and transparent treatment with xylene.

### 2.8 Serum cytokine level measurement

Following the sacrifice of the mice, whole blood was drawn and subsequently centrifuged for 15 min at 6,000 × *g*. The harvested serum was then preserved at −80°C. Shanghai Jianglai Industrial Share Ltd. (Shanghai, China) supplied the ELISA kit that was utilized to measure serum levels of IL‐10, IL‐17, IL‐1β, and TNF‐α, using the manufacturer's instructions for the kit.

### 2.9 Immunofluorescent staining for CD68 and CD86

The levels of CD68 and CD86 expression in the colon tissue of mice from different experimental groups were examined using immunofluorescent staining. A 5‐μm‐thick section of colon tissue, embedded in paraffin, was placed on a positively charged slide for analysis. The slides were kept in xylene to be deparaffinized, repeated twice for 10 min each, followed by rehydration using a gradient decreasing ethanol concentration. Antigen retrieval was achieved by subjecting the slides to citrate buffer treatment in a microwave oven. To prevent nonspecific binding, the tissue section was blocked with blocking reagents for 20 min. Subsequently, the slides were rinsed with PBS and then exposed to primary antibodies targeting CD68 and CD86, as outlined in Table 1, and left to incubate overnight at 4°C. Following further washes, the slides were exposed to secondary antibodies conjugated with fluorochromes fluorescein isothiocyanate (FITC) (Proteintech, Wuhan, China) for 1 h at room temperature under dark conditions. To visualize the cell nuclei, 4′,6‐diamidino‐2‐phenylindole (DAPI) staining was conducted for 5 min, followed by three subsequent washes with PBS for 5 min each. Finally, the slides were mounted and examined under a microscope.

**TABLE 1:** Antibodies used in IHC, IF, and Western blotting.

Antibody target	Antibody type	Antibody dilution	Company
Occluden	Monoclonal	1:5000	Proteintech
ZO‐1	Polyclonal	1:200	Bioss
Mucin‐2	Polyclonal	1:2000	Proteintech
8‐OHG	Polyclonal	1:500	Bioss
CD86 and CD68	Polyclonal	1:2000	Proteintech
FOXP3	Polyclonal	1:2000	Proteintech
TLR4	Polyclonal	1:500	Proteintech
IL‐6	Polyclonal	1:1000	Proteintech
IKK‐β	Polyclonal	1:1000	Bioss
IkB‐α	Polyclonal	1:1000	Bioss
NF‐κB	Polyclonal	1:1000	Bioss

### 2.10 Quantification of mRNA expression levels using real‐time PCR

Colon tissue samples were employed for total RNA extraction utilizing Triazole reagents acquired from Thermo Fisher Scientific, followed by storage at −80°C. Quantification of messenger RNA (mRNA) levels in the colon was conducted using RT‐qPCR (reverse transcription‐quantitative polymerase chain reaction). Complementary DNA (cDNA) was synthesized using a reverse transcriptase kit, and RT‐qPCR was performed using the SYBR Green RT‐qPCR kit from Takara (Japan). All the primers used in this work were synthesized by Sangon Biotech Shanghai China and are listed in Table 2. Triplicate analyses were conducted for each sample, and relative gene expression was calculated and evaluated using LineGene 9660 system software and GraphPad Prism to compare differences among various experimental groups.

**TABLE 2:** List of primer sequences employed for assessing mRNA expression level.

Gene	Forward primer	Reverse primer
CR	GATTCCTGAGGCTCCAACACAC	ACAGTGTAGCCCTTGTGCAGAC
IFN‐ɤ	CAGCAACAGCAAGGCGAAAAAGG	TTTCCGCTTCCTGAGGCTGGAT
IL‐18	ACCAGGAGCCATATCCACGGATG	TGTTCTTACAGGAGAGGGTAGAC
IL‐4	CATTGCTGGTCCAGTCTGCTTCG	GGTGTTCTTCGTTGCTGTGAGGAC
TGF‐β	CATTGCTGGTCCAGTCTGCTTCG	TGGTGAATGACAGTGCGGTTATGG
β‐Actin	ATCGCTGCGCTGGTCG	GTCCTTCTGACCCATTCCCA

### 2.11 Immunohistochemistry for GATA3, FOXP3, IL‐6, and 8‐OHG in colon

To investigate the expression of GATA‐binding protein 3 (GATA3), forkhead box P3 (FOXP3), and IL‐6 expression in colon tissue, immunohistochemical staining was performed. Initially, a 5‐μm‐thick section of colon tissue was placed on slides with positively charged postparaffin embedding. The slides underwent deparaffinization in xylene, followed by rehydration through a gradient of decreasing ethanol concentrations. Subsequently, a 3% hydrogen peroxide (H_2_O_2_) solution was applied for 20 min to block endogenous peroxidase activity. Antigen retrieval was facilitated by heating the slides in an antigen retrieval buffer using a microwave oven. Following this, the tissue sections were left to incubate overnight at 4°C with primary antibodies specific to GATA3, FOXP3, and IL‐6. After the primary incubation, the slides were rinsed with PBS and then exposed to a secondary antibody for 1 h. Visualization of antibody binding was achieved through staining with a 3,3′‐diaminobenzidine (DAB) substrate. Finally, the slides were washed, fixed, and examined under a light microscope at various magnifications. Cells with immunolabeling were identified through random inspection of each slide three times across diverse fields.

### 2.12 Western blot analysis for expressed proteins

In the present study, protein extraction was conducted utilizing radioimmunoprecipitation assay (RIPA) lysis buffer. Subsequently, the extracted proteins were subjected to be separated via sodium dodecyl sulfate–polyacrylamide gel electrophoresis (SDS–PAGE), employing gradient gels ranging from 8% to 12% concentration. Following electrophoretic separation, the proteins were transferred onto a polyvinylidene difluoride (PVDF) membrane, facilitating subsequent immunodetection analyses. The transferred membrane underwent a blocking step with 5% skimmed milk for 2 h. After blocking, the membrane was thoroughly washed with Tris‐buffered saline supplemented with Tween 20 (TBST) to eliminate residual blocking agents and other contaminants. Following washing, the membrane, alongside the primary antibody, was incubated at a refrigerated temperature (4°C) overnight. Following incubation with primary antibody, the membrane was subjected to rigorous washing to remove unbound primary antibody molecules. Subsequently, the membrane was exposed to a secondary antibody conjugated with an appropriate detection moiety for 1 h at ambient temperature. Following incubation with the secondary antibody, the membrane underwent additional washing steps to remove excess secondary antibody and any residual contaminants. Finally, protein bands were visualized through the application of an enhanced chemiluminescent (ECL) substrate, and subsequent imaging was performed utilizing a gel documentation system.

### 2.13 Statistical analysis

For statistical analysis of the study, GraphPad Prism 9.5 was used. A one‐way analysis of variance (ANOVA) and subsequent post hoc multiple comparison tests were used to assess the statistical significance.

## 3 RESULTS

### 3.1 Determination of CPH molecular weight distribution

**FIGURE 1:**
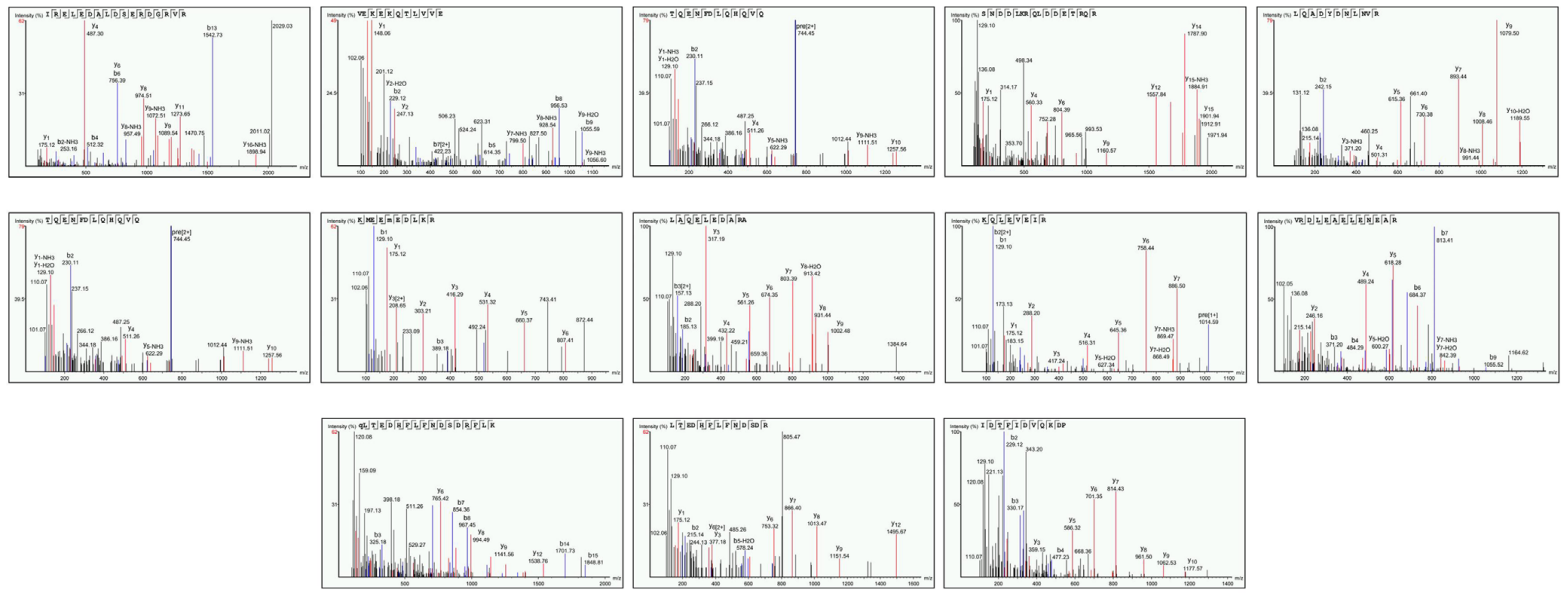
Liquid chromatography–mass spectrometry (LC–MS) analysis of conch peptides hydrolysate. The figure represents the results of LC–MS analysis conducted on conch peptides hydrolysate. Thirteen distinct spectra were observed, representing different peptides with diverse amino acid compositions and lengths published previously (Ullah et al., 2023).

The hydrolysate was prepared using trypsin enzyme for digestion and freeze‐dried using a lyophilizer machine. To examine the molecular makeup of the hydrolysate liquid chromatography–mass spectrometry (LC–MS) was utilized to see the protein, peptides, and amino acid composition in hydrolysate, as depicted in Figure 1. The results revealed 13 different separate spectra that represent unique peptides with varying lengths and amino acid composition, as shown in Figure 1.

### 3.2 Fourier‐transform infrared spectroscopy (FTIR)

**FIGURE 2:**
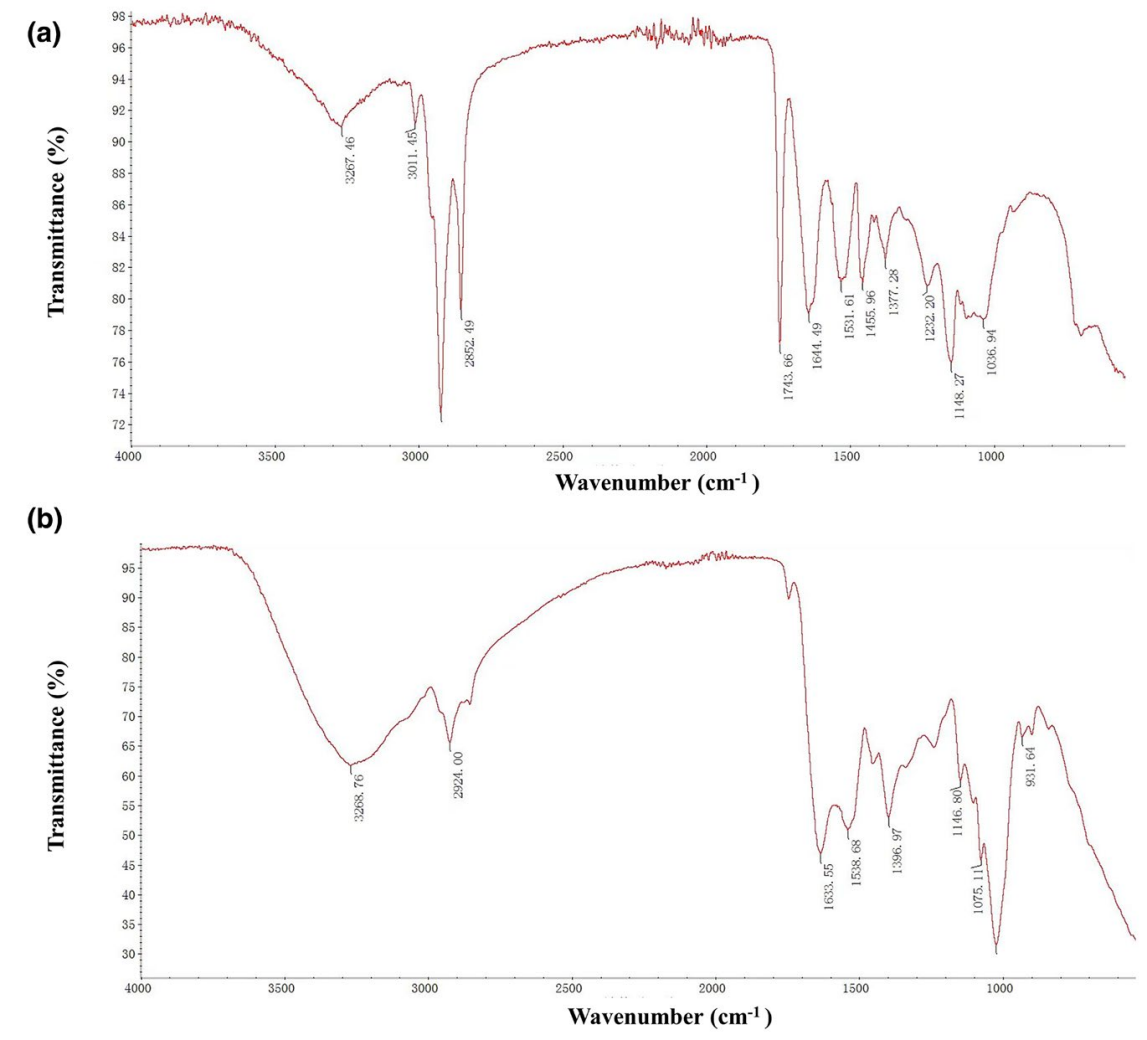
The FTIR spectra of the crude sample and peptide hydrolysate were analyzed, revealing distinct peak appearances: (a) FTIR spectrum of the crude sample and (b) FTIR spectrum of the CPH sample.

To examine the chemical structure and functional group, peptides hydrolysate and crude sample were analyzed using the Fourier‐transform infrared spectroscopy (FTIR). The analysis revealed the distinct differences between the crude sample and peptide hydrolysate. The crude sample shows broad and sharp peaks at 2922 and 2852 cm^−1^ representing the aliphatic hydrocarbon, while other prominent peaks at 1455 and 1377 cm^−1^ indicate the presence of methyl and methylene groups. Peak appearance at 1743 cm^−1^ indicates the presence of carbonyl compound, similarly at 1644 and 1531 cm^−1^, showing the amide group and aromatic compound, respectively, as shown in Figure 2a. However, in the case of peptides hydrolysate, results exhibited noticeable changes. The peaks appear at 3268 cm^−1^, confirming the formation of peptides. The appearance of the peak at 1021 cm^−1^ shows the presence of glycosidic peptides, as depicted in Figure 2b. This chemical structure transformation highlights the successful enzymatic digestion of crude sample into peptides.

### 3.3 Scanning electronic microscopy (SEM)

**FIGURE 3:**
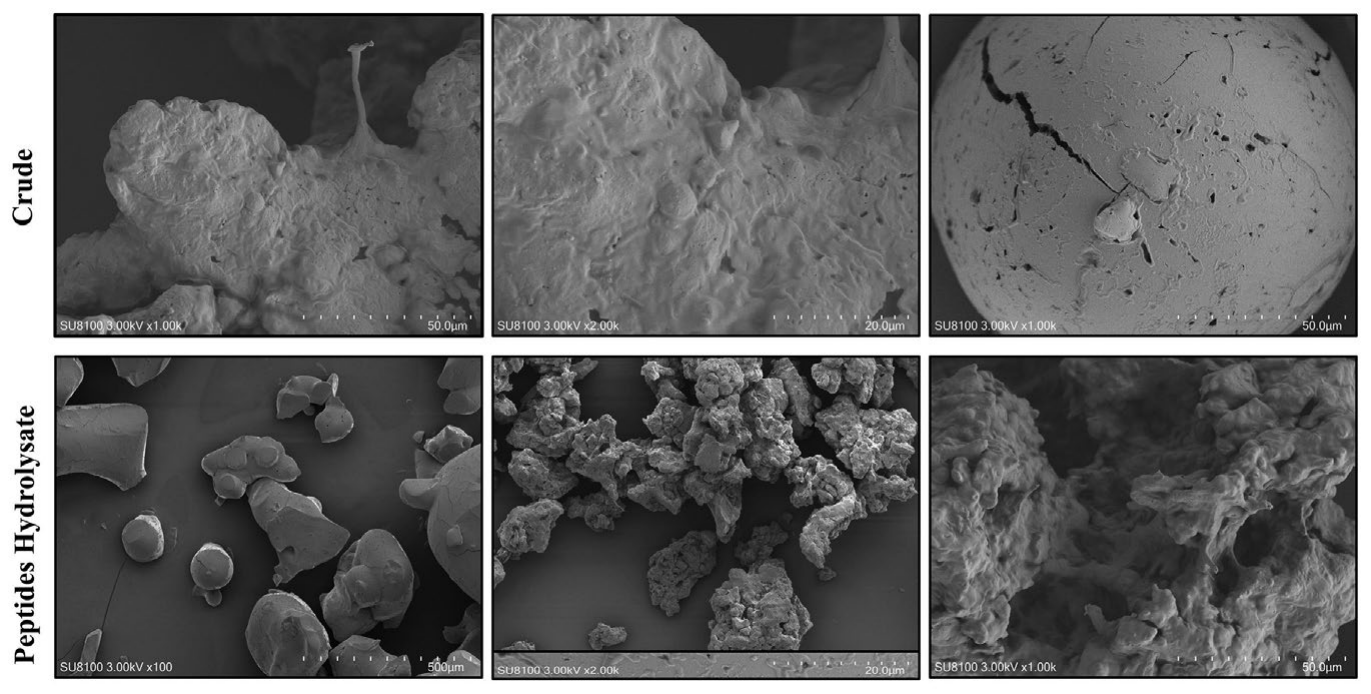
Scanning electronic microscopy of peptides hydrolysate and crude (without enzyme digestion) samples at different magnifications.

To investigate the morphological changes induced by enzymatic hydrolysis of the peptide's hydrolysate and crude sample, scanning electron microscopy was performed. The SEM images revealed significant differences in the structure morphology at different magnifications. In the case of the crude sample (without enzyme digestion), the observed structures exhibited a relatively large size indicating the presence of aggregated or conglomerated molecules. This appearance shows the presence of large molecule entities in the crude sample. While the peptide hydrolysate displayed a distinct transformation in structure. At higher magnification, the peptide hydrolysate shows a fragmented appearance, suggesting the breakdown of larger molecules into smaller molecules. The results revealed the useful enzymatic hydrolysis of conch protein, leading to the formation of peptide fragments. The SEM analysis provides visual evidence of the morphological changes that occurred during enzymatic hydrolysis, confirming the conversion of breaking of large molecules into small fragments, as shown in Figure 3.

### 3.4 CPH alleviates pathological manifestation in DSS‐induced colitis mice

**FIGURE 4:**
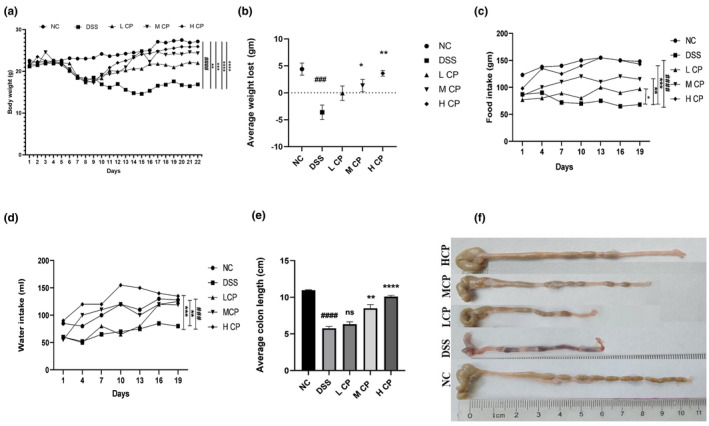
The therapeutic effects of CPH were prominently observed in mice with DSS‐induced colitis, effectively mitigating pathological symptoms. The evaluation encompassed various parameters: (a) alterations in body weight, (b) average weight loss, (c) food consumption, (d) intake of water, (e) average colon length, and (f) comparison of colon length. Statistical analyses were performed in comparison to the negative control, represented as ### *p* < .001, #### *p* < .001. Comparisons against the DSS group were denoted as **p < .01, ***p < .001, ****p < .0001, and ns, non‐significant.

After subjecting mice to 15 days of treatment with CPH for DSS‐induced colitis, an evaluation was performed to assess its therapeutic efficacy. In Figure S1, the Disease Activity Index (DAI) parameters are assessed through the evaluation of body weight loss, stool consistency, and rectal bleeding. Following administration of DSS via drinking water, a notable decrease in body weight was observed across all mice, as depicted in Figure 4a. When comparing the DSS group of mice to the control group, a significant drop in body weight was noted. The administration of CPH, on the other hand, produced a noticeable improvement in body weight after 15 days. The effectiveness of CPH varied depending on the dosage, with medium and high doses (MCP and HCP) showing a dose‐dependent enhancement in body weight, as shown in Figure 4a. The group treated with DSS (dextran sodium sulfate) experienced substantial weight loss because of rectal bleeding and severe diarrhea, unlike the normal control group (NC) and the groups receiving treatment. Figure 4b illustrates that supplementation with medium and high doses of CPH alleviated body weight loss. DSS intake is known to affect water and food consumption, with the DSS group displaying reduced intake of food and water, indicating compromised health status. However, administration of CPH led to an increase in these consumption parameters, as demonstrated in Figure 4c,d. Additionally, the DSS group's colon length was noticeably shorter than that of the negative control group. Conversely, as shown in Figure 4e,f, the administration of medium and high doses of CPH led to a considerable elongation of the colon, whereas the administration of low dose of CPH did not produce a significant increase.

### 3.5 CPH improves histomorphology of colon, small intestine, and spleen

**FIGURE 5:**
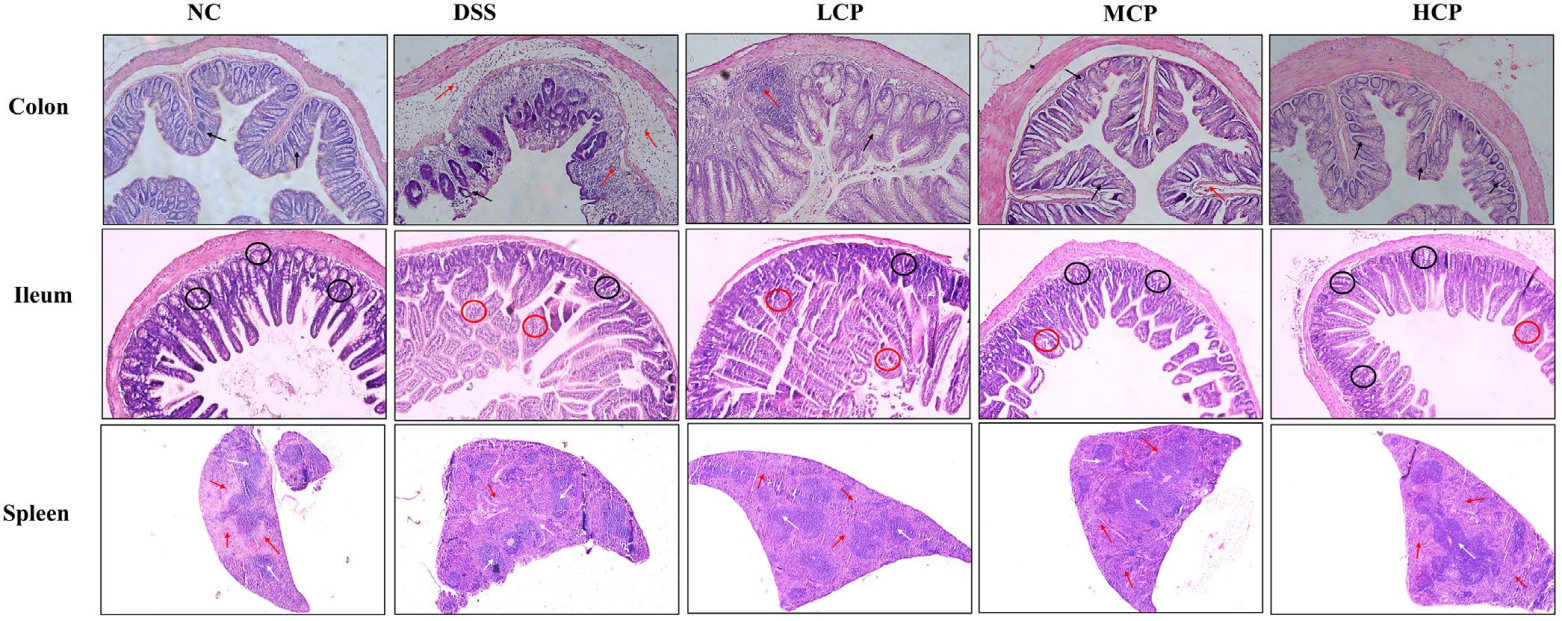
Hematoxylin and eosin (HE) staining, CPH supplementation mitigated the histopathological alterations. CPH healing effect on the colon (black arrow indicates goblet cells and the red arrow indicates inflammatory cells), small intestine (black circle indicates goblet cells and the red circle indicates inflammation), and spleen (white arrows: white pulp; red arrows: red pulp) on DSS‐induced colitis mice, magnification (10×).

Hematoxylin and eosin (HE) staining was used to count the goblet cells and examine the histomorphology of the colon, small intestine, and spleen to assess the effect of CPH therapy on DSS‐induced colitis mice. The colon and small intestine of the normal group of mice displayed well‐defined, densely compacted columnar epithelium with clear borders between the mucosal and submucosal layers, according to the results of hematoxylin and eosin (HE) staining. There was very little inflammation visible, and there were a lot of goblet cells. In contrast, the DSS‐treated group showed notable abnormalities, characterized by severe tissue damage, and inflammation, the crypt structure appeared distorted and shallow with few numbers of goblet cells and disrupted architecture. However, the histological results exhibited that CPH administration improved damaged colon tissue in a dose‐dependent manner. The low‐dose CPH showed moderate improvement, with reduced inflammation and tissue damage compared to the DSS group. The medium‐ and high‐dose CPH treatment groups showed significant improvement, with near‐normal histomorphology, minimal inflammation, and restoration of tissue integrity, as shown in Figure 5. Additionally, the DSS group displayed disrupted splenic architecture characterized by an undefined margin between white and red pulp, which reveals abnormal distribution and organization of the different compartments within the spleen. However, in the normal control group, the spleen shows normal histology with intact white and red pulp. The white pulp, which contains immune cells and is involved in immune response, maintained its distinct structure and organization. CPH administration exhibited a therapeutic effect improving spleen morphology. Specifically, CPH administration resulted in the restoration of the defined margin between the white and red pulp, indicating a more organized splenic architecture, as shown in Figure 5.

### 3.6 Effect of conch peptides hydrolysate on mucin production

**FIGURE 6:**
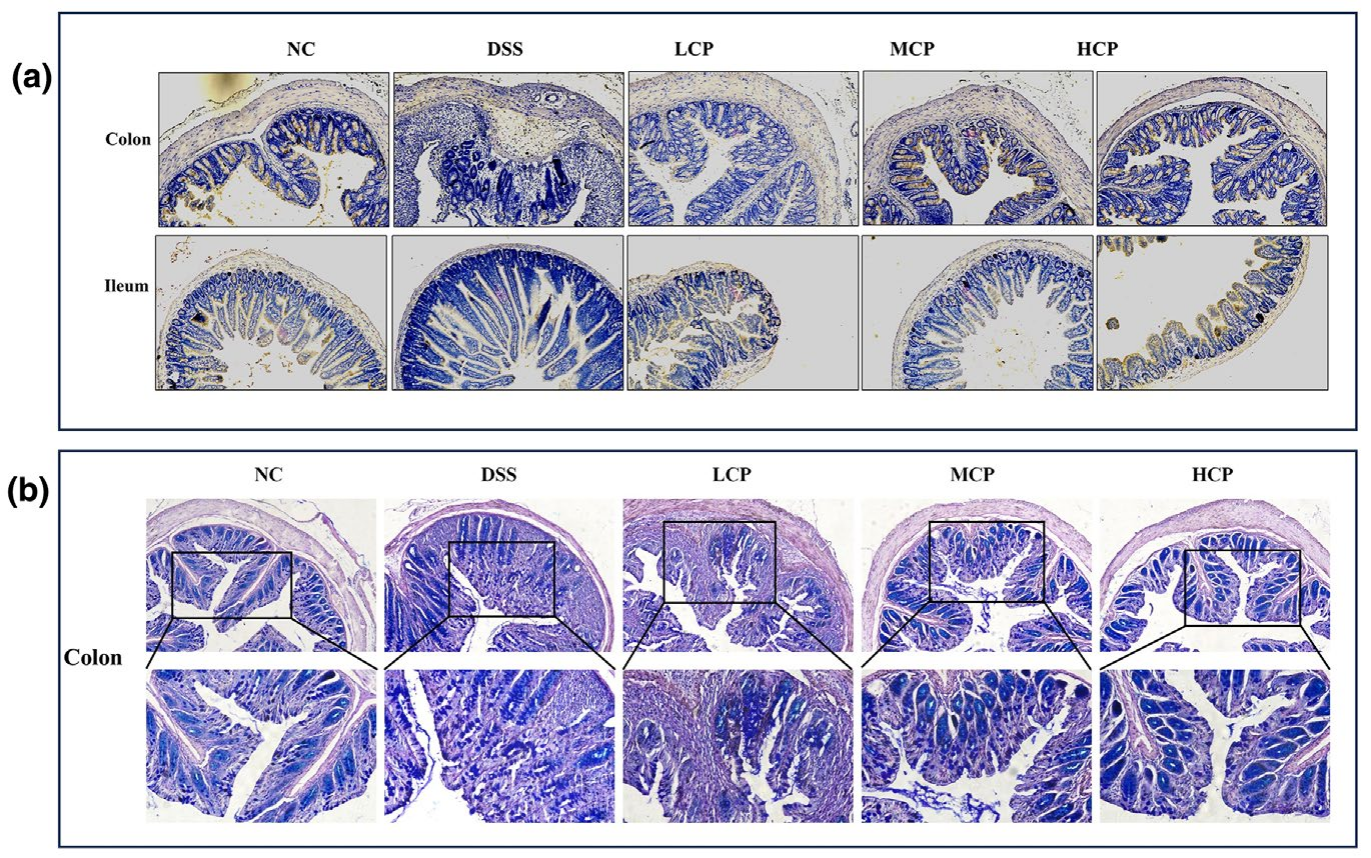
Sea conch peptide hydrolysate (CPH) administration enhances the expression of Mucin‐2 and increases the production of goblet cells. (a) Immunohistochemistry of colon and small intestine of different groups' magnification (10×). (b) Alcian blue‐periodic acid–Schiff (AB‐PAS) staining represents the images of the colon section of different groups' magnification upper (10×) and lower (20×).

Immunohistochemistry and AB‐PAS staining were performed to assess the ameliorative impact of conch peptide hydrolysate on the expression of mucin in the context of DSS‐treated mice. Results revealed that, in the normal control group higher mucin expression was observed, indicating the presence of healthy and functional goblet cells and mucin layer in the small intestine and colon. In contrast, the DSS‐alone group showed significantly reduced expression of Mucin‐2 in the colon and small intestine, indicating the disruption in the production of mucin secretion. The decrease in expression of Mucin‐2 is consistent with the known pathophysiological changes associated with colitis, including mucosal damage and inflammation. However, CPH treatment demonstrated a dose‐dependent increase in Mucin‐2 production. In the CPH‐supplemented groups, the high‐dose group led to a more pronounced increase in Mucin‐2 expression, both in the colon and small intestine, as illustrated in Figure 6.

### 3.7 CPH regulates serum cytokines

**FIGURE 7:**
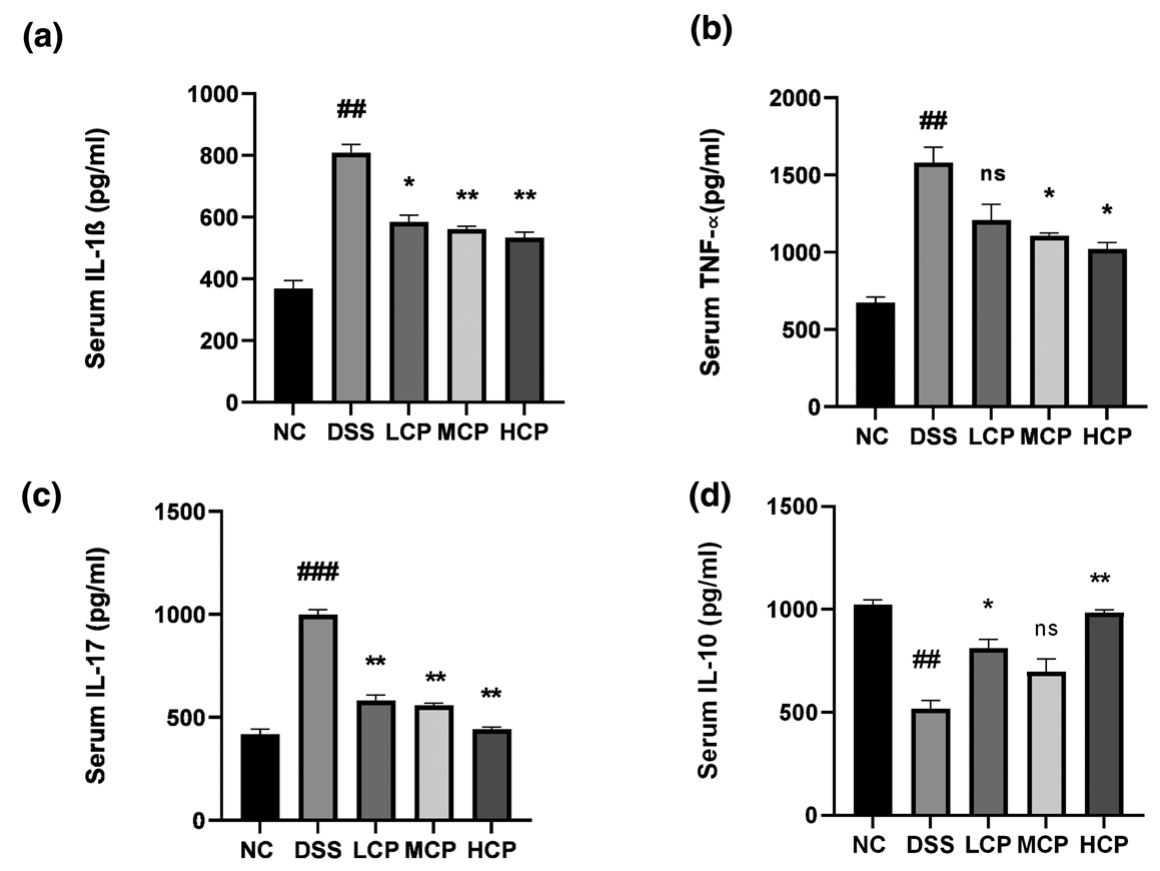
Sea conch peptide hydrolysate (CPH) treatment affects the serum cytokine level. (a) IL‐1β, (b) TNF‐α, (c) IL‐17, and (d) IL‐10. Comparison to normal control; ## *p* < .01, ### *p* < .001, comparison to DSS group *p < .05, **p < .01, ***p < .01, and ****p < .01.

To assess the potential therapeutic efficacy of CPH in a DSS‐induced colitis mouse model, ELISA analysis was utilized to quantify the levels of various cytokines in the serum. The results indicated upregulation of pro‐inflammatory cytokines IL‐1β, IL‐10, TNF‐α, and IL‐17. Specifically, the group treated with DSS exhibited significantly elevated levels of IL‐1β, TNF‐α, and IL‐17 compared to the NC group (*p* < .01, *p* < .01, and *p* < .001, respectively). However, administration of CPH for 15 days resulted in a notable reduction in the expression of these cytokines in a dose‐dependent manner, particularly at higher doses. Figure 7 illustrates a substantial decrease in the expression of IL‐1β (*p* < .05, *p* < .01, and *p* < .01, respectively), TNF‐α (*p* < .05, *p* < .05), and IL‐17 (*p* < .01, *p* < .01, and *p* < .01, respectively), as depicted in Figure 7a–c. Additionally, CPH demonstrated anti‐inflammatory activity by upregulating the expression of IL‐10 in the treatment groups. Conversely, Figure 7d illustrates a decreased expression of IL‐10 in the group treated solely with DSS (*p* < .01).

### 3.8 CPH Administration modulates the expression of GATA3, FOXP3, and IL‐6 in the colon

**FIGURE 8:**
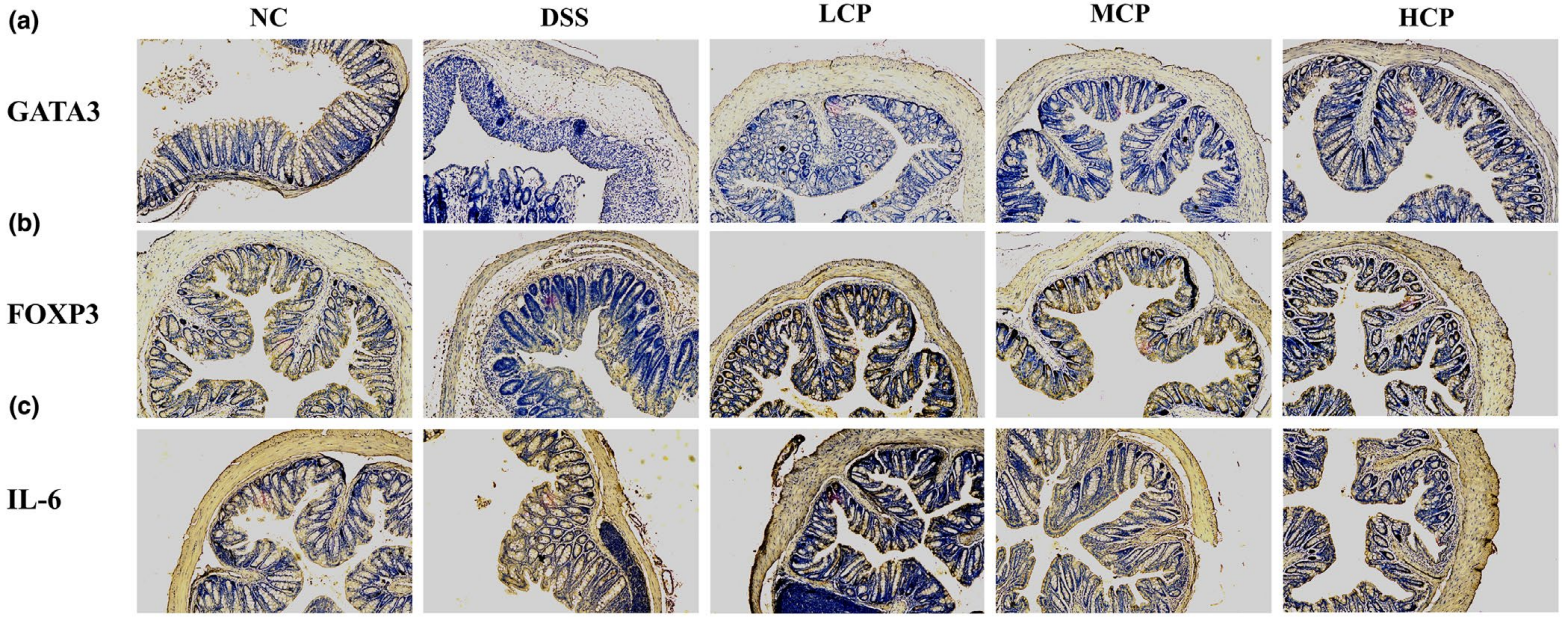
Immunohistochemical analysis of GATA3, FOXP3, and IL‐6 expression in the colonic tissue. (a) Expression level of GTAT3. (b) Expression level of FOXP3. (c) Expression level of IL‐6 in colonic tissue. Picture magnification (10×).

To assess the immunomodulatory effects of CPH, immunohistochemistry was employed to examine the expression levels of GATA3, FOXP3, and IL‐6 in the colons of mice with DSS‐induced colitis. The results revealed distinct expression patterns of these immune markers across different experimental groups. Compared to the DSS‐treated group, the negative control group exhibited higher expression levels of FOXP3 and GATA3. Conversely, the DSS‐treated group displayed lower expression of these markers, indicating potential disruption in regulatory T cell (Treg) function and immune dysregulation associated with colitis. However, following the administration of CPH, a noticeable dose‐responsive increase in the expression levels of FOXP3 and GATA3 was observed, particularly with high doses of CPH. This suggests a potential improvement in intestinal immune homeostasis in response to CPH treatment, as illustrated in Figure 8a,b. Additionally, the expression level of IL‐6 was assessed. The DSS‐treated groups exhibited a significant increase in IL‐6 expression compared to the NC group. However, in the CPH treatment groups, a notable decrease in IL‐6 expression was observed, indicating the anti‐inflammatory activity of CPH, as depicted in Figure 8c.

### 3.9 CPH treatment modulates the CD68 and CD86 expression in colon

**FIGURE 9:**
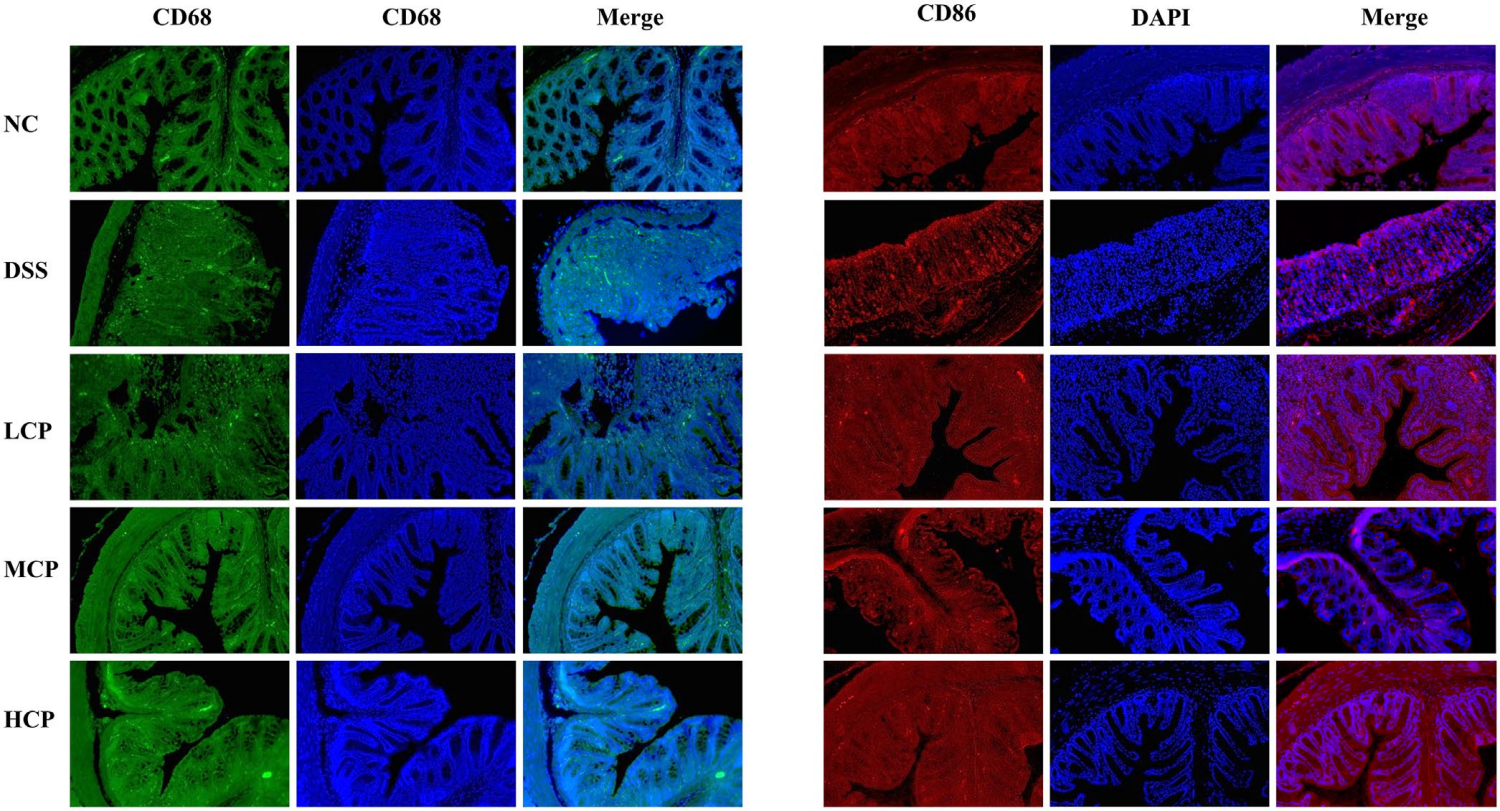
Immunofluorescent staining of CD68 and CD86 in colonic tissue of different experimental group mice. Expression of CD68 and CD86. Picture magnification (20×).

Immunofluorescent analysis was utilized to assess the expression levels of CD68 and CD86 in the colon tissue of mice from different experimental groups. The objective was to explore the immunomodulatory impact of peptide hydrolysate (CPH) on DSS‐induced colitis in mice. As illustrated in Figure 9, increased levels of CD68 and CD86 expression were noted in the colon tissue of the DSS group in comparison to the NC group. However, subsequent supplementation with CPH resulted in a significant dose‐dependent decrease in the expression levels of these markers. Particularly, higher doses of CPH showed a more pronounced effect on decreasing CD68 and CD86 expression, suggesting a potential decrease in immune cell activation, as indicated in Figure 9.

### 3.10 CPH regulate the expression of pro‐ and anti‐inflammatory cytokine levels in colon

**FIGURE 10:**
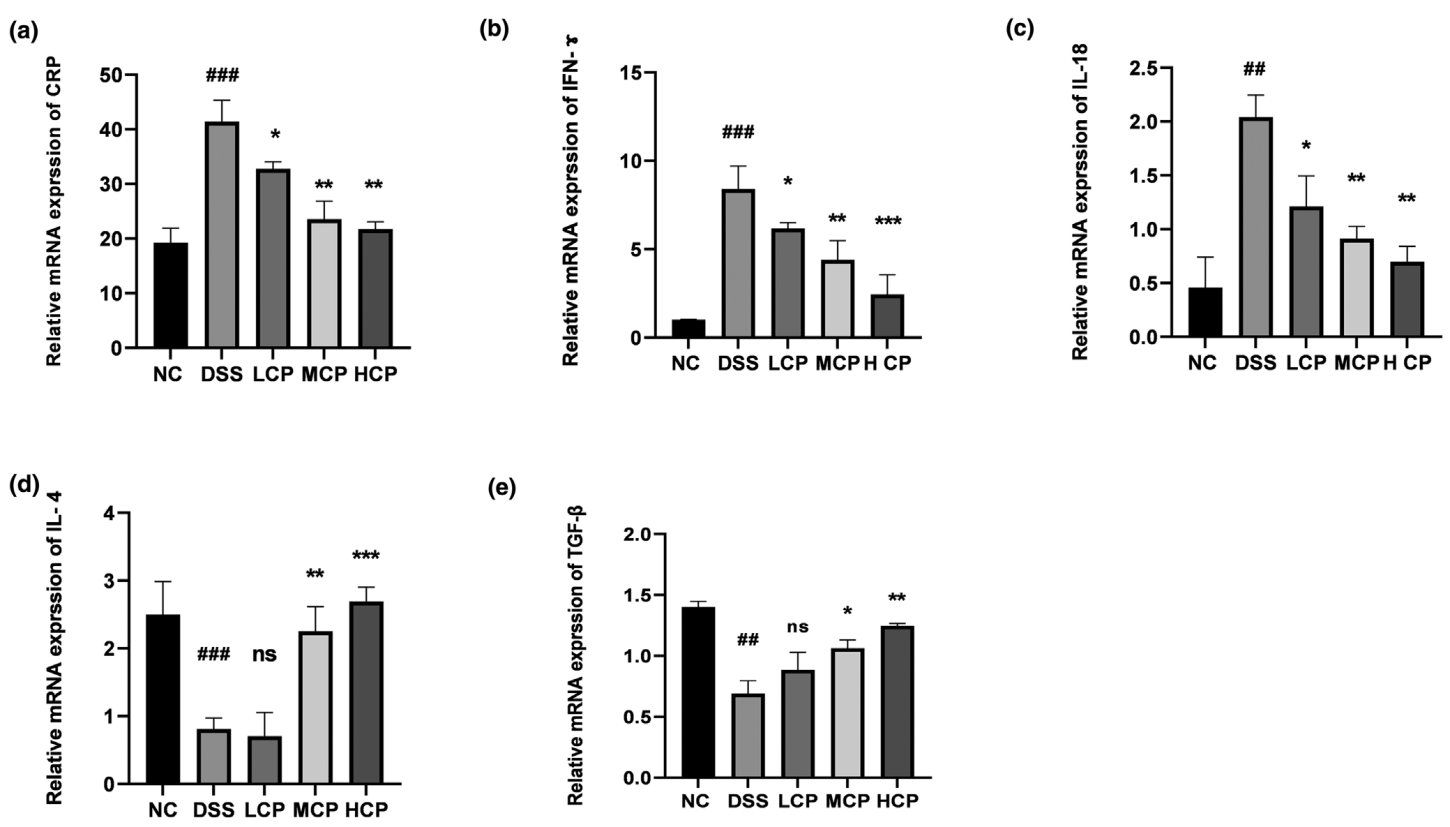
Messenger RNA (mRNA) expression levels of inflammatory markers in the colon. (a) C‐reactive protein, (b) IFN‐ɤ, (c) IL‐18, (d) IL‐4, and (e) TGF‐β. The expression was standardized with β‐actin expression. And results reflected: comparison to normal control ## *p* < .01, ### *p* < .001, comparison to DSS group *p < .05, **p < .01, ***p < .001, ns, non‐significant.

Further evaluation of gene expression level in colon tissue of DSS‐induced colitis mice was performed using RT‐qPCR analysis to assess the effects of CPH therapy. Relative mRNA expression levels of pro‐inflammatory (interferon gamma (IFN‐ɤ) and interleukin 18 (IL‐18)), anti‐inflammatory (interleukin 4 (IL‐4) and transforming growth factor beta (TGF‐β)), and C‐reactive protein (CRP) were assessed, as depicted in Figure 10. CRP expression was substantially higher in the DSS group than in the normal control group (*p* < .001). Figure 10a illustrates the significant dose‐dependent decrease in CRP expression that occurred upon the administration of CPH as a therapy (*p* < .05, *p* < .01, and *p* < .01, respectively), in the same order. Additionally, there was a significant difference in the expression levels of IFN‐ɤ (*p* < .001) and IL‐18 (*p* < .01) between the DSS group and the normal control group. As seen in Figure 10b,c, CPH administration reduced the production of IL‐18 and IFN‐ɤ, especially at the high dose (*p* < .001, *p* < .01). Furthermore, the DSS‐treated group showed a reduction in the mRNA expression levels of the anti‐inflammatory cytokines TGF‐β (*p* < .01) and IL‐4 (*p* < .001). On the other hand, as shown in Figure 10d,e, CPH treatment, especially at high dosages, dramatically elevated the expression of TGF‐β (*p* < .01) and IL‐4 (*p* < .001).

### 3.11 CPH modulates inflammatory signaling pathways and increases TJS expression

**FIGURE 11:**
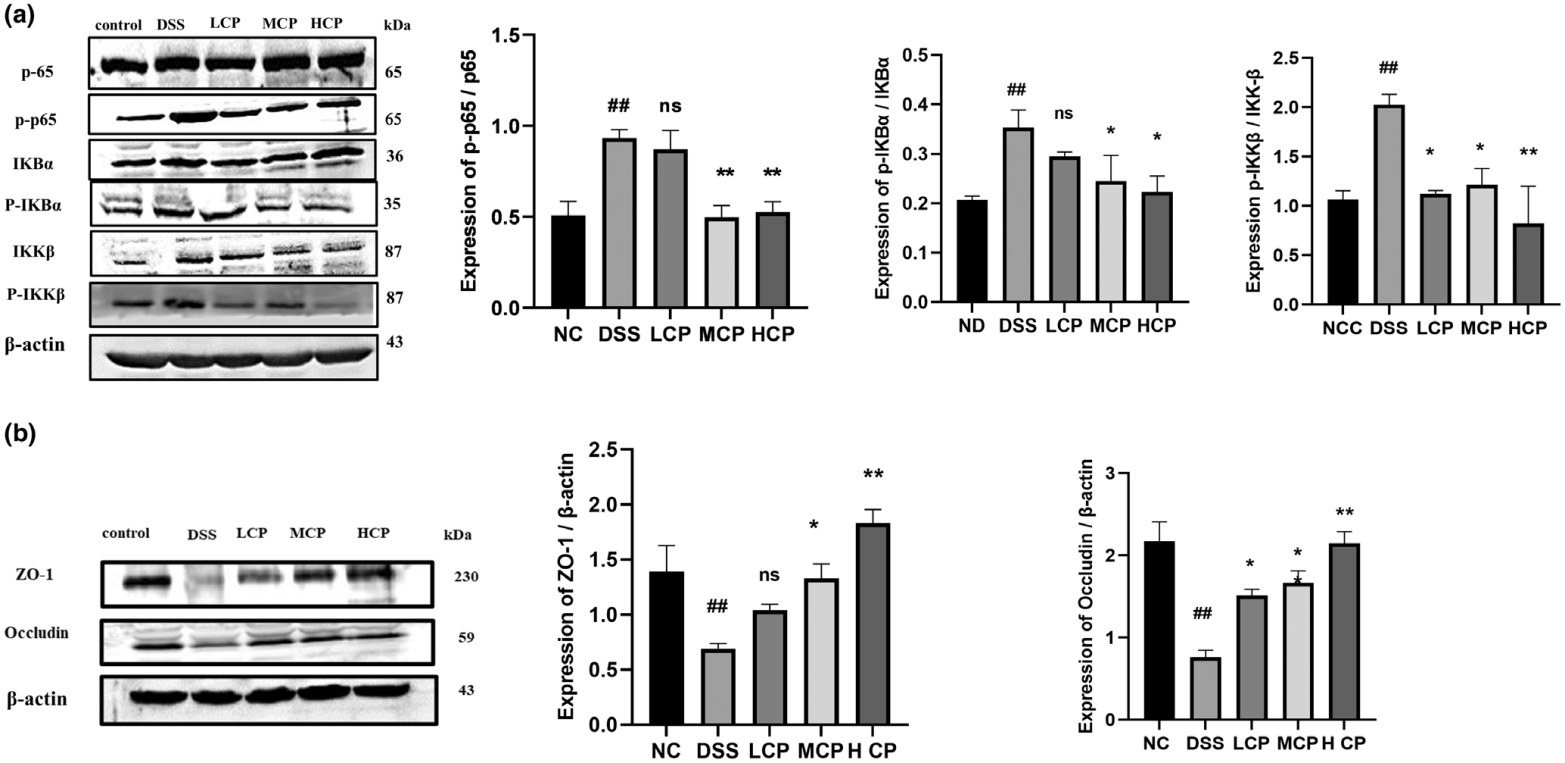
Differential modulation of colitis‐associated pathway and tight junction expression by CPH treatment. (a) Western blot images showing the expression of proteins p65, p‐p65, p‐IκB‐α, p‐IκB‐α, IKK‐β, and p‐IKK‐β. (b) The relative expression levels of tight junction proteins ZO‐1 and Occludin in colonic tissue, with β‐actin as a loading sample. Comparison to normal control ## *p* < .01, comparison to DSS group; *p < .05, **p < .01, ns, non‐significant.

**FIGURE 12:**
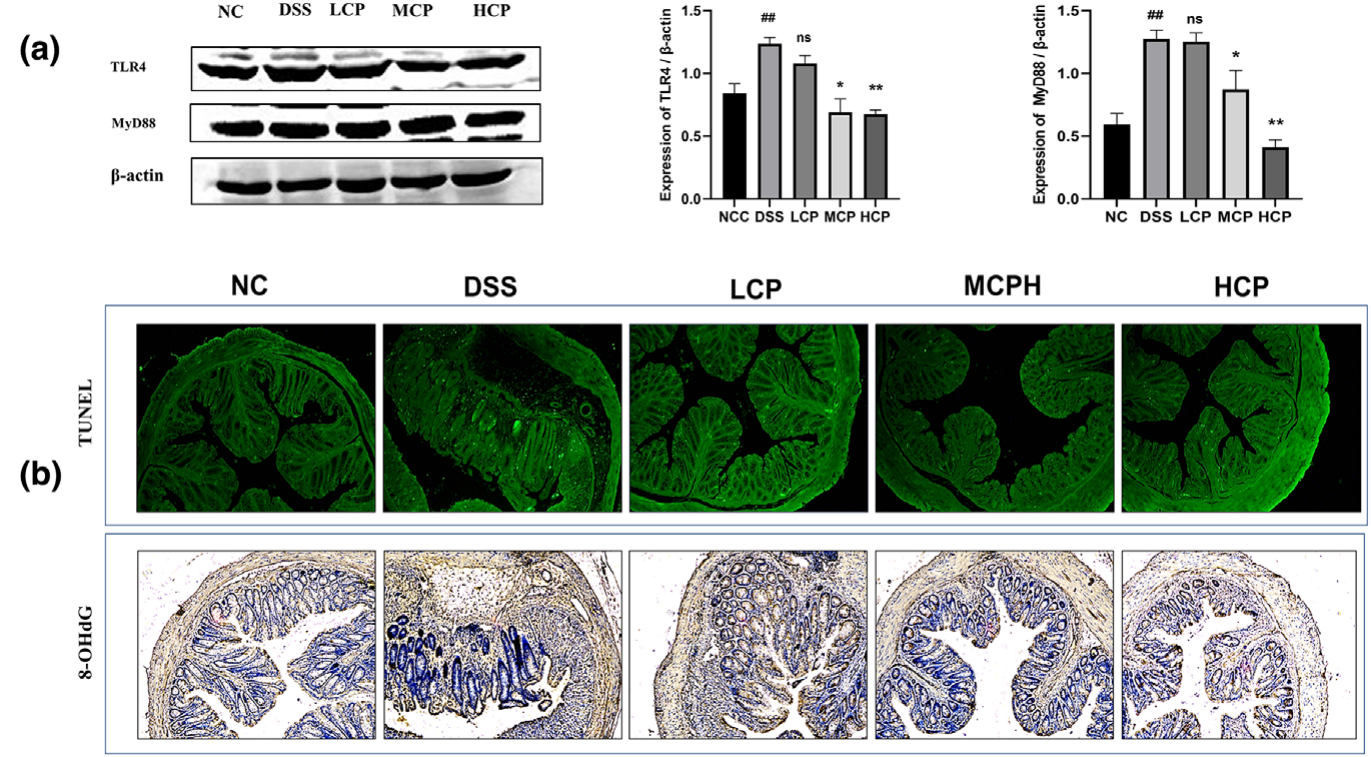
(a) Western blot images showing the relative expression of TLR4 and MyD88. β‐Actin served as the internal control. Bar graph representing the relative expression of each protein analyzed by ImageJ. Data reflected as ±SEM ### *p* < .01 versus normal control, versus DSS *p < .05, **p < .01, and ns, non‐significant. (b) Sea conch peptide hydrolysate (CPH) reduces oxidative stress and cell apoptosis. TUNEL assay (green, fluorescent assay) and immunohistochemical staining for 8‐OHG of colon section; the picture shows the histological features (magnification 10×).

To explore the immunoregulatory effects of CPH on inframammary signaling pathways, we analyzed several key factors. In the DSS‐treated group, there was a notable increase in the phosphorylation of NF‐κB p‐65 (*p* < .01), IKB‐α (*p* < .01), and inhibitory kappa B kinase beta (IKK‐β) (*p* < .01) compared to the normal control group (Figure 11a). However, following CPH treatment, particularly at a high dose for 15 days, the phosphorylation of phospho (p)‐NF‐κB (*p* < .01), phospho (p)‐IκB‐α (*p* < .05), and phospho (p)‐IKK‐β (*p* < .01) significantly decreased, indicating downregulation of these signaling molecules. Additionally, we assessed the impact of CPH on tight junction proteins after DSS treatment. The expression of Occludin and Zonula occludens 1 (ZO‐1) was markedly reduced in the DSS‐treated group compared to the normal group (*p* < .01 for both) (Figure 11b). Nevertheless, CPH treatment, especially at high doses, significantly increased the relative expression level of ZO‐1 and Occludin (*p* < .01) compared to the DSS‐treated group, suggesting a protective effect of CPH on tight junction integrity. Moreover, we analyzed the Toll‐like receptor 4 (TLR4) pathway. The protein expression levels of key components in the TLR4 pathway were altered in the DSS group (Figure 12a). TLR4 expression (*p* < .01) and myeloid differentiation primary response 88 (MyD88) (*p* < .01) were found to be higher in the DSS‐alone group compared to the normal group. Nonetheless, treatment with hydrolysate, specifically at a high dose, significantly lowered the expression of TLR4, MyD88, and p‐NF‐κB (*p* < .05) respectively, suggesting an inhibitory effect of CPH on the TLR4 pathway.

### 3.12 CPH supplementation reduces the oxidative stress and apoptosis

The TUNEL (terminal deoxynucleotidyl transferase dUTP nick‐end labeling) assay was utilized to assess oxidative stress levels in the colon and investigate the potential of CPH in mitigating DSS‐induced colitis in mice. Findings revealed that the DSS‐treated group exhibited a higher number of TUNEL‐positive cells compared to the normal control group. However, following administration of CPH, a dose‐dependent reduction in TUNEL‐positive cells was observed across the low, medium, and high dosage groups. Additionally, the prevalence of 8‐hydroxyguanosine (8‐OHG)‐positive cells was elevated in the DSS‐treated group, whereas CPH treatment led to a significant decrease in these cells within the colonic mucosa of mice across the treatment groups, as depicted in Figure 12b.

## 4 DISCUSSION

Inflammatory bowel disease (IBD) is a long‐lasting group of inflammatory illnesses of the digestive system affecting the gastrointestinal tract, primarily consisting of ulcerative colitis and Crohn's disease. The condition is characterized by an abnormal immune response in the gut (Miao et al., 2015). The precise mechanism of IBD is not fully understood, but it is thought to be involved in different factors to cause this condition. In animal models, DSS is frequently used, which causes experimental colitis. DSS administration in drinking water disrupts the integrity of the intestinal barrier, which can cause an inflammatory response. DSS‐utilized induced colitis models have played an important role in studying the mechanisms underlying colitis, investigating the immune system function and their involvement in the pathogenesis of inflammatory bowel disease (IBD), and identifying potential sources of treatment. Natural products including polysaccharides, plant extract, mushroom peptides, and mushrooms have shown promise in alternative therapy in various inflammatory responses, such as gastric ulcer, colitis, and chronic gastritis (Liu et al., 2008; Miao et al., 2015; Muszyńska et al., 2018; Wang et al., 2015). Consumer attitudes toward health and foods are changing worldwide. People want to consume health‐beneficial and safe products. This concern about healthy and safe products is leading to a higher demand for them in the market (Kim et al., 2010; Yoon et al., 2009). Recently, researchers proposed a suggestion that food contains peptides with biological activity. These bioactive peptides from food sources have been found with various applications in human health. Peptides are reported to have different activities, such as reducing high blood pressure and acting as anticoagulants, immune modulators, anti‐microbial peptides, and antioxidants (Najafian & Babji, 2012). In the current work, we investigated the immune regulatory effect of the enzymatic hydrolysate derived from sea conch proteins. In our findings, we demonstrated that CPH possesses potential immune regulatory properties. Moreover, we used different techniques to characterize our peptide hydrolysate.

In our study, we found that the trypsin enzyme was the most effective for the enzymatic digestion of conch meat. We used liquid chromatography–mass spectrometry (LC–MS), to characterize the peptide hydrolysate to identify and analyze different peptides present in the hydrolysate. LC–MS results revealed a diverse range of peptides with a distinctly different molecular weight and unique amino‐acid combinations. Notably, our finding aligns with a previously published work that utilized MALDI‐TOF (time‐of‐flight spectrometry) analysis to identify different peptide spectra from the shrimp peptides hydrolysate (Khan et al., 2022).

Fourier‐transform infrared spectroscopy has become a popular technique for structural characterization of proteins and peptides. Its extensive spectral range covers vibrational frequencies of various chemical groups in both polypeptide chains and other compounds. This allows for detailed analysis and structural characterization of protein interaction with lipids, nucleic acids, drugs, and other molecules providing structural information on all components that generate absorbance bands in specific spectral regions (Tatulian, 2013). FTIR analysis results exhibited that crude sample and peptide hydrolysate have notable changes in the functional groups. The characteristic peaks in the crude sample represent the presence of aliphatic hydrocarbon, organic compounds, carbonyl compounds, and aromatic compounds. The functional group characteristics changed after enzymatic digestion. In the peptide's hydrolysate sample, additional peaks represent the presence of peptides as well as amide groups, which provides further insight into the molecular composition and chemical structure of peptides present in hydrolysate samples. These results are supported by a previously published study by (Lee et al., 2022) who observed similar peaks for peptides present in the sample of collagen hydrolysate extracted from Alaska pollock skin. Our results are consistent with those of another study by (Abadía‐García et al., 2021) who reported similar results on peptides from whey proteins, which further prove the validity of our structure analysis using FTIR spectroscopy.

Scanning electronic microscopy is important in modern pharmaceutical technology for the structural characterization of complex drug delivery systems and individual compounds like adjuvants, drugs, and impurities, utilizing techniques, such as transmission electron microscopy (TEM), SEM, cryogenic electron microscopy (cryo‐EM), and analytical EM methods (Klang et al., 2013). Under electron microscopy, crude sample and peptide hydrolysate were visualized. Results revealed that the structural morphology of digested hydrolysate was different than that of the crude sample. The structure of crude sample (without enzyme digestion) observed exhibited a relatively large size indicating the presence of aggregated or conglomerated molecules. This appearance shows the presence of large molecule entities in the crude sample. While the peptides hydrolysate displayed a distinct transformation in structure. At higher magnification, the peptides hydrolysate shows a fragmented appearance, suggesting the breakdown of larger molecules into smaller molecules. The structure and morphology of the crude sample and peptides hydrolysate have been examined and reported by (Lee et al., 2022) using scanning electronic microscopy to study collagen hydrolysate extracted from Alaska pollock skin. Also, our findings are in parallel with previously published work by (Rozi et al., 2018) who used SEM to observe the morphological characteristics of larger proteins and peptides. Their findings highlighted structural differences between these components, similar to our observation.

In this study, we used 2.5% DSS to induce ulcerative colitis in mice by continuous administration in drinking water. The DSS‐treated group displayed high loss in body weight, shrinkage of the colon, and low food and water intake. However, treatment with CPH exhibits a remedial effect and alleviates the severity of these symptoms. Similar findings were reported in a study (Li et al., 2022) where *Seriola quinqueradiata* hydrolysate showed a treatment effect in dextran sodium sulfate mice.

Consuming DSS causes histological changes through goblet cell death, decreased tight junction expression, mucus depletion, and epithelial cell apoptosis. High intestinal permeability, blood in the stool, and a high death rate are examples of more severe symptoms (Han et al., 2021; Zhang et al., 2020). In our study, we observed histological alterations in the colon, small intestine, and spleen. In the histology of the colon and small intestine of the DSS‐treated group, notable abnormalities were observed and characterized by severe tissue damage, inflammation, and distorted crypt structure. However, CPH treatment heals all the pathological manifestations caused by DSS. To compare and demonstrate similarity with these results, our findings align with those of (Kanwal et al., 2020) showing that DIP treatment alleviated the severity of colitis, histopathological alterations and gut epithelial integrity were ameliorated, while inflammatory reaction and oxidative stress were improved.

In the development and pathophysiology of IBS, pro‐inflammatory cytokines have a significant role. Several research works have highlighted the important role these cytokines play in driving the immune system and dysregulation and chronic inflammation observed in IBS. Numerous cytokines and chemokines have been shown to express themselves highly in colitis. The development of colitis has been directly associated with inflammatory cytokines, such as IL‐6, IL‐17, TNF‐α, and interleukin 23 (IL‐23) (Fina & Pallone, 2008; Randhawa et al., 2014). In the present study, we measured the expression of different cytokines (pro‐ and anti‐inflammatory cytokines) from serum and colonic tissue. The DSS group exhibited an increased expression of pro‐inflammatory cytokine levels and a decreased level of anti‐inflammatory cytokine level. Previously published studies support our results (Diling et al., 2017; Li et al., 2016).

Regulatory T (Treg) cells operate through mechanisms that control immune responses. Central to their suppressive function is the stable expression of the transcription factor FOXP3, which collaborates with other transcription factors to activate anti‐inflammatory responses and suppress pro‐inflammatory ones (Vent‐Schmidt et al., 2014). To further investigate the CPH effect on intestinal immunity, we further target important immune markers, such as FOXP3, GATA3, and IL‐6. Our results revealed lower expression of GATA3 and FOXP3 in the colon of DSS‐treated group but higher expression of IL‐6. Conversely, the group treated with CPH exhibits notable changes in the expression of these immunological markers. CPH increases the production of FOXP3 and GATA3 in the colon and suppresses the production of IL‐6, showing its promise of therapeutic potential. Similar results were seen by (Park et al., 2018), showing the treatment effect of *Lactobacillus acidophilus* on intestinal inflammation.

Under normal physiological health conditions, macrophage phenotypes maintain a dynamic equilibrium to uphold intestinal immune balance. Elevated macrophage presence in the colon lamina propria is a hallmark of the inflammatory lesions (Kühl et al., 2015). Imbalance in the intestinal homeostasis due to abnormal M1/M2 macrophage polarization is implicated in the development of IBD (Choi et al., 2020). Our study also explores the effect of CPH on adaptive immune response. Our results exhibited high expression levels of CD68 and CD86 positive cells in the DSS‐alone group. While CPH administration reinstates the intestinal immune response through the reduction in the expression of these cells. A similar effect on these immune cells has been observed by (Ardizzone et al., 2023) studying the effect of *Ulva pertusa* in DSS‐induced colitis.

Nuclear factor kappa B (NF‐κB) proteins are vital transcription factors involved in regulating various physiological processes, including immune response, apoptosis, cell proliferation, inflammation, and malignant transformation. Abnormal NF‐κB activity, whether due to mutations or disruptions in its transcription activity, has been linked to the development of leukemia, lymphomas, and solid tumors (Rayet & Gélinas, 1999). In the current study, considering the treatment potential of CPH in ameliorating DSS‐induced colitis mice, we extended our investigation to target inflammatory‐related signaling pathways associated with IBD. According to previously published research, the TLR4 and NF‐κB signaling pathways are crucial for the development of colitis (Li et al., 2014, 2016). Our results explore the treatment effect of CPH on ulcerative colitis via dephosphorylation of NF‐κB, TLR4, and MyD88, respectively, in CPH treatment groups.

In colitis conditions, there is an observation of both an increase in cell proliferation and an increase in cell apoptosis. This phenomenon has been attributed to a recurrence–remission cycle (Iwamoto et al., 1996; Strater et al., 1997). The hyperproliferation and abnormal distribution in UC have been suggested as a biomarker for an elevated risk of developing colorectal cancer (Lipkin, 1988). Because highly regulated processes are crucial for tissue remolding and repair after injury. In our study, we used 2.5% DSS orally for 7 days in drinking water, which caused different symptoms like diarrhea, bleeding, and tissue damage. We extend our investigation to check the anti‐apoptotic effect of CPH in DSS‐induced colitis. Our examination revealed a higher number of TUNEL‐positive cells in the DSS‐treated group. However, CPH supplementation causes a notable reduction in TUNEL‐positive cells. Moreover, the expression of 8‐OHG was found to be higher in the DSS group, while upon CPH treatment the 8‐OHG cells indicating oxidative stress were reduced. Similar results were reported by (Park et al., 2018) who used *Lactobacillus acidophilus* species for the treatment of DSS‐induced colitis.

## 5 CONCLUSION

Sea conch peptide hydrolysate (CPH) shows promising effects in alleviating colitis symptoms. CPH administration led to an elongation of the colon and improved body weight food, and water intake. The expression levels of serum and colon pro‐inflammatory cytokines were reduced, and anti‐inflammatory cytokine levels were elevated, followed by higher expression of anti‐inflammatory markers such as FOXP3 and GATA3, indicating improved intestinal immune regulation. Additionally, targeting inflammatory‐related signaling pathways, such as NF‐κB and TLR4, were found to be regulated with CPH treatment. CPH also exhibits anti‐apoptotic activity, reduces oxidative stress, and enhances the expression of tight junction protein. These findings suggest that conch peptide hydrolysate can potentially improve intestinal immune homeostasis and holds promise for using therapeutic potential for managing colitis and dysregulated response.

## AUTHOR CONTRIBUTIONS

**Hidayat Ullah:** Methodology (equal); conceptualization (equal); investigation (equal); formal analysis (equal); writing – original draft (equal); data curation (equal). **Yamina Alioui:** Methodology (equal). **Muhsin Ali:** Methodology (equal). **Sharafat Ali:** Methodology (equal). **Nabeel Ahmed Farooqui:** Methodology (equal). **Nimra Z. Siddiqui:** Investigation (equal); methodology (equal). **Duaa M. Alsholi:** Methodology (equal). **Muhammad Ilyas:** Methodology (equal). **Mujeeb U. Rahman:** Methodology (equal); software (equal). **Yi Xin:** Conceptualization (equal); project administration (equal); software (equal); supervision (equal); visualization (equal); writing – review and editing (equal). **Liang Wang:** Conceptualization (equal); funding acquisition (equal); investigation (equal); project administration (equal); supervision (equal); writing – review and editing (equal).

## FUNDING INFORMATION

This research was supported by National Nature Science Foundation of China. Grant Number (Grant Nos. 31600614 and 82072953) and Chinese Scholarship Council (CSC) grant number (2019BSZ007223).

## CONFLICT OF INTEREST STATEMENT

The authors declare no conflict of interest.

## ANIMAL AND HUMAN RIGHTS STATEMENT

All procedures involving mice were approved by the Dalian Medical University Animal Ethics Committee (Protocol Number: 202310247) and followed Institutional Animal Care and Use Committee (IACUC) guidelines. No human subjects were involved.

## Supporting information


Data S1.

## Data Availability

The original data for this work are available upon email request to the corresponding author.
