# Supplementary material for: Sea conch (Rapana venosa) peptide hydrolysate regulates NF‐κB pathway and restores intestinal immune homeostasis in DSS‐induced colitis mice
Source: Food Sci Nutr. 2024 Sep 25;12(12):10070–86. doi: 10.1002/fsn3.4410 (PMC11666983; doi:10.1002/fsn3.4410)
Supplement: Supplementary file 1 — Data S1. [file FSN3-12-10070-s001.docx]

**Sea conch (*Rapana Venosa*) Peptide Hydrolysate Regulate NF-κB Pathway and Restore Intestinal Immune Homeostasis in DSS-Induced Colitis Mice**

**Hidayat Ullah^1^, Yamina Alioui^1^, Muhsin Ali1^1^, Sharafat Ali^2^, Nabeel Ahmed Farooqui^1^, Nimra Z. Siddiqui^1^, Duaa M. Alsholi^1^, Muhammad Ilyas^1^,** **Mujeeb U. Rahman^1^, Yi Xin^1^ and Liang Wang^3^*.**

**1: Supplementary Information**

## Supplementary Figure

**Figure S1**. Disease Activity Index (DAI) measurement**.** DAI scores were calculated based on weight loss, diarrhea, and rectal bleeding. Results reflected as vs **NC** #### p < 0.0001, vs **DSS**: ** p < 0.01, *** p < 0.01.

**1.2 Supplementary Table**

**Table S1.** Disease Activity index measurement criteria

| **Score** | **Weight loss %** | **Stool consistency** | **Rectal bleeding** |
| --- | --- | --- | --- |
| **0** | None | Normal | Negative |
| **1** | 1-5 |  |  |
| **2** | 5-10 | Loose stool | Positive |
| **3** | 10-20 | Diarrhea | Positive |
| **4** | *˃* 20 | Diarrhea | Gross bleeding |
